# Isolated Foveal Hypoplasia: Tomographic, Angiographic and Autofluorescence Patterns

**DOI:** 10.1155/2012/864958

**Published:** 2012-07-31

**Authors:** Ágata Mota, Sofia Fonseca, Ângela Carneiro, Augusto Magalhães, Elisete Brandão, Fernando Falcão-Reis

**Affiliations:** ^1^Department of Ophthalmology, Hospital S. João, Alameda Prof. Hernâni Monteiro, 4200-319 Porto, Portugal; ^2^Department of Ophthalmology, Hospital Vila Nova de Gaia/Espinho, Rua Conceição Fernandes, 4434-502 Vila Nova de Gaia, Portugal; ^3^Faculdade de Medicina, Universidade do Porto, Alameda Prof. Hernâni Monteiro, 4200-319 Porto, Portugal

## Abstract

*Purpose*. To report clinical aspects, tomographic, angiographic, and autofluorescence patterns of two cases of isolated foveal hypoplasia. *Methods*. Foveal hypoplasia was found in a 23-year-old male patient and in a 64-year-old woman with impaired visual acuity of unknown etiology that remained unchanged for years. *Results*. In the first case, spectral-domain optical coherence tomography (SD-OCT) showed reduced foveal pit and continuity of inner retinal layers in the fovea. Photoreceptor layer had a normal thickness centrally. The foveal avascular zone (FAZ) was absent in the flourescein angiogram (FA). Fundus autofluorescence showed reduced foveal attenuation of autofluorescence. In the second patient, there was the same pattern in SD-OCT, with normal aspect in FA and only a slightly reduced foveal attenuation of autofluorescence. *Conclusion*. OCT, as a noninvasive and quick method, is helpful in the diagnosis of foveal hypoplasia. FA and fundus autofluorescence were less sensitive.

## 1. Introduction

Foveal hypoplasia has been described a lack of foveal depression with continuity of all neurosensory retinal layers in the presumed foveal area [1–4]. It is well known in aniridia, albinism, microphthalmus, achromatopsia, and retinopathy of prematurity [1–3, 5, 6]. Foveal hypoplasia as an isolated finding is rare [2]. In recent years, optical coherence tomography (OCT) has been described as a quick and useful tool to confirm the suspected diagnosis [1, 2].

We report clinical aspects, tomographic, angiographic, and autofluorescence patterns of two cases of isolated foveal hypoplasia.

## 2. Material and Methods

Clinical records, tomographic, angiographic, and autofluorescence patterns of two patients with isolated foveal hypoplasia were reviewed. Fundus autofluorescence imaging, spectral-domain optical coherence tomography (SD-OCT), and flourescein angiogram (FA) were obtained with the HRA + OCT, Heidelberg Engineering, Germany.

## 3. Results

**Figure 1:**
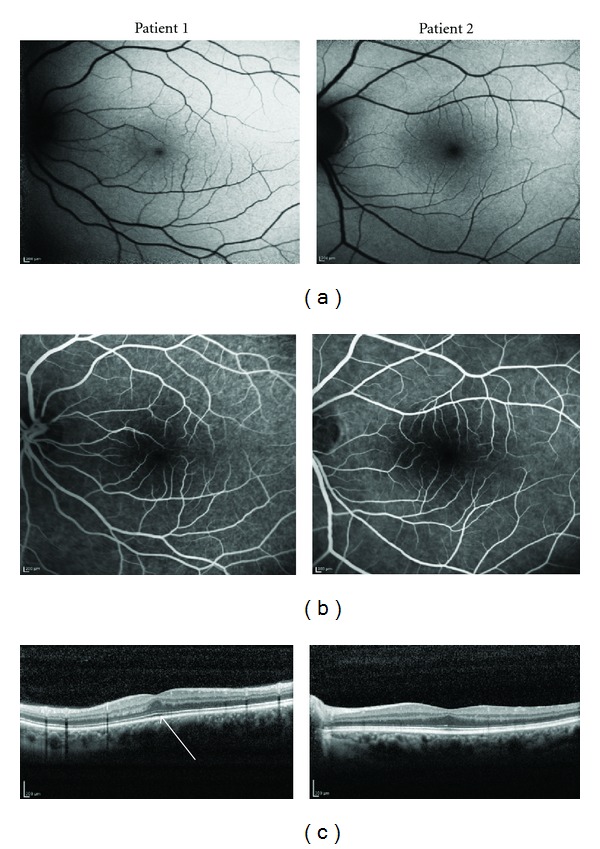
(a) Fundus autofluorescence imaging did not show the typical foveal darkening due to absorption of the excitation light by macular pigment in patient 1. Patient 2 had only a slightly reduced foveal attenuation of autofluorescence. (b) The foveal avascular zone was absent in the flourescein angiogram in patient 1. Patient 2 had a normal aspect in fluorescein angiography. (c) SD-OCT showed reduced foveal pit and continuity of inner retinal layers in both patients. Photoreceptor layer had a normal thickness centrally in patient 1 (arrow).

Healthy 23-year-old, caucasian male patient presented with best corrected visual acuity (BCVA) of 8/10 in right eye and 6/10 in left eye. There was no nystagmus, iris transillumination, or aniridia. The fundus had a normal pigmentation, with a reduced foveolar reflex. SD-OCT showed reduced foveal pit and continuity of all inner retinal layers (Figure 1(c)). Photoreceptor layer had a normal thickness centrally. The foveal avascular zone (FAZ) was absent in the FA (Figure 1(b)). Fundus autofluorescence imaging did not show the typical foveal darkening due to absorption of the excitation light by macular pigment (Figure 1(a)).

The second patient is a 64-year-old caucasian woman, with a decreased vision since childhood, with no progression throughout her life. BCVA was 1/10 in right eye and hand motions in left eye. There was no nystagmus, iris transillumination, or aniridia. The fundus had a normal pigmentation, macula showed slightly mottled pigment irregularities, and there was a reduced foveolar reflex. In this patient we report the same pattern in SD-OCT, with normal aspect in FA (Figures 1(b) and 1(c)). Fundus autofluorescence had only a slightly reduced foveal attenuation of autofluorescence (Figure 1(a)).

## 4. Discussion

Fundoscopic aspect in foveal hypoplasia may vary from unremarkable except for absent foveal reflexes to slightly mottled pigment irregularities [2], as we found in these two cases. Typically, FA shows absence of FAZ, which could be seen in case 1, but not in case 2. In the literature we find that foveal hypoplasia is associated with a broad range of visual acuities from 20/20 to 20/400 [6].

Some authors tried to find an association between visual acuity and foveal morphology, but the role of foveal depression was not confirmed as a prognostic factor [6]. Neither the foveal avascular zone nor a pit is critical for the postnatal lengthening of cones or spatial packing that takes place to make higher visual resolution. Foveal cone specialization can be preserved both anatomically and functionally despite the absence of a foveal depression. *Fovea plana* is a term recently suggested to denote only the anatomic lack of a foveal pit with no functional implications [3, 6]. This explains why some patients may have good visual acuity despite lack of normal foveal depression, as observed in the first patient. Recently, Thomas et al. developed a grading system based on presence or absence of foveal pit and widening of the outer nuclear layer and outer segment at the fovea. The grading system was developed based on the stage at which foveal development was arrested and helped to provide a prognostic indicator for VA and was applicable in a range of disorders associated with foveal hypoplasia [7].

The normal thickness of the photoreceptor layer found in patient 1 at the presumed foveal center is also present in healthy eyes [2]. This may be a sign of lesser anatomic alteration in a subset of patients with foveal hypoplasia, and this was not seen in the second case, which showed worse visual acuity.

Issa et al. suggest that an intact foveal anatomy appears to be related to physiologic macular pigment storage in the neurosensory retina. They speculated that macular pigment density might correlate with the anatomical and functional integrity of the fovea in patients with foveal hypoplasia [2]. In both cases we present, there is a slightly (case 2) or markedly (case 1) reduced of foveal attenuation of fundus autofluorescence by macular pigment. But this finding was more evident in patient 1 who had better visual acuity. In our opinion, the visual outcome is probably determined by multifactorial aspects besides foveal anatomy and amount of pigment deposition.

In both cases OCT confirmed the diagnosis. It may also become useful in predicting visual outcome. Both, in the literature and as is this 2 cases, we observe that there is a wide variability of clinical manifestations (fundal aspect, visual acuities), angiography and autofluorescence patterns. But there is a common manifestation to all cases, which is SD-OCT pattern with reduced or absent foveal pit and continuity of inner retinal layers.

## 5. Conclusion

SD-OCT, as a noninvasive and quick method, is helpful in the diagnosis of foveal hypoplasia [1, 2], especially in those patients with impaired or subnormal vision of unknown etiology. Clinical aspect, fluorescein angiography, and fundus autofluorescence patterns, although important, seem less sensitive to make the diagnosis.
